# Psychosocial impact of perinatal loss among Muslim women

**DOI:** 10.1186/1472-6874-12-15

**Published:** 2012-06-18

**Authors:** Rosnah Sutan, Hazlina Mohd Miskam

**Affiliations:** 1 Community Health Department, UKM Medical Centre, Jalan Yaakob Latif, Bandar Tun Razak, 56000, Cheras, Kuala Lumpur, Malaysia

**Keywords:** Perinatal loss, Healthcare provider, Psychosocial, Muslim

## Abstract

**Background:**

Women of reproductive age are vulnerable to psychosocial problems, but these have remained largely unexplored in Muslim women in developing countries. The aim of this study was to explore and describe psychosocial impact and social support following perinatal loss among Muslim women.

**Methods:**

A qualitative study was conducted in a specialist centre among Muslim mothers who had experienced perinatal loss. Purposive sampling to achieve maximum variation among Muslims in relation to age, parity and previous perinatal death was used. Data was collected by focus group discussion and in-depth unstructured interview until the saturation point met. Sixteen mothers who had recent perinatal loss of wanted pregnancy, had received antenatal follow up from public or private health clinics, and had delivery in our centre participated for the study. All of them had experienced psychological difficulties including feelings of confusion, emptiness and anxiety over facing another pregnancy.

**Results:**

Two out of sixteen showed anger and one felt guilt. They reported experiencing a lack of communication and privacy in the hospital during the period of grief. Family members and friends play an important role in providing support. The majority agreed that the decision makers were husbands and families instead of themselves. The respondents felt that repetitive reminder of whatever happened was a test from God improved their sense of self-worth. They appreciated this reminder especially when it came from husband, family or friends closed to them.

**Conclusion:**

Muslim mothers who had experienced perinatal loss showed some level of adverse psychosocial impact which affected their feelings. Husbands and family members were the main decision makers for Muslim women. Health care providers should provide psychosocial support during antenatal, delivery and postnatal care. On-going support involving husband should be available where needed.

## Background

The loss of a baby either during pregnancy or after delivery holds great significance for parents and their close family members. This loss may be perceived as a loss of the parent’s future hopes and of their potential for fulfilling their dreams. Evidence-based showed that perinatal loss significantly increases anxiety in a subsequent pregnancy and often produces feelings of guilt [1-3]. The significance of pregnancy, and the consequences of perinatal loss, differs depending on personal as well as cultural values. Schaap, et al. (1997) conducted a study on emotional impact among parents experienced intrauterine death or neonatal death showed that 50% of the respondents did not share or discuss their emotions and most of them did not feel the loss of their own child [4]. A study performed by Leon stated that women with perinatal loss expressed that they had nothing to see or grieve the death of their babies. Leon described that there were no social conventions to confer personhood on the lost child and, crucially, that there was no recognition by family, friends, and medical caregivers that a major loss had occurred [5]. However, the incongruent grieving between partners was more pronounced in the intrauterine death and often led to long-term emotional disturbances and psychosocial problems [5].

Psychosocial intervention for women with perinatal loss needs to be tailored to cultural needs, but the problems of Muslim women are often poorly addressed, particularly in non-Muslim or multiethnic communities. According to statistics (2004) showed that Muslim males and females in Great Britain had the highest rates of reported ill health in year 2001 and Muslims had the highest rates of disability[6]. The report by the Pew Forum on Religion & Public Life (2010) showed that 62.1% of global Muslim population hailed from Asia-Pacific region [7]. Malaysia is a multi-ethnic country with four main ethnic groups: ‘Bumiputera’, Chinese, Indian and others. The ‘Bumiputera’ are a group of Malaysian citizens consisting of Malays and indigenous people and forms the majority group in Malaysia. The Malaysian population Census 2010 divided the Malaysian population into Bumiputera 67.4%, Chinese 24.6%, Indian 7.3% and others 0.7% [8]. Based on the 2000 census, the Malays who are predominantly Muslims, comprised 65.1% of the population (estimated at 23.3 million) and that 63.5% of the total population lived in urban areas [8]. Forte and Horton-Deutsch (2005) mentioned in their reviews that fundamentals of Islam and its religious practices is related to reproductive process in Muslim women [9]. These authors stated that social isolation, homesickness and feeling unsupported were associated with higher rates of depression. Although Islam is made up of numerous different ethnic groups, there are certain fundamental beliefs that all Muslims share regardless of culture in Malaysia and the rest of the world. Islam requires the total acceptance and compliance with the teachings of Allah as seen in the Qur’an to his last prophet Mohammad [10].

According to annual report of stillbirths and neonatal deaths in 2005 reported that for 1998–2002, the largest proportion of all perinatal deaths were classified as normally formed, macerated stillbirths [11]. An earlier study performed in Malaysia showed that perinatal loss is highest among Indians, followed by the Bumiputera[12]. Sutan (2010) in her study showed that according to the Edinburgh Postnatal Depression Scale (EPDS) score, 53.2% of the respondents had suffered a psychosocial impact [13]. The authors observed that significant inverse relationship between psychosocial impact after perinatal loss and support from friends (P = 0.019) [13]. Life experience and major life events may shape the behaviour of the individual and indirectly affect their health and people surrounding them. Using phenomenology approach, rich textured descriptions of life experiences can be explored [14]. Finlay (2009) said that this approach is suitable to explicate this intentionality to do with the directedness of participants’ consciousness by exploring what and how they are experiencing [14].

The role of support groups after perinatal loss is well established and well known in United Kingdom, United States of America, Australia through the Stillbirths and Neonatal Death Society (SANDS) group and others but they cease to exist in Malaysia [13]. Côté-Arsenault D and Freije MM (2004) found that support groups for a parent with perinatal loss helped members recognize their commonalities, remember their earlier babies who died, develop caring relationships, and learn new coping skills [15]. Several large population based studies have been conducted in many countries related to perinatal loss but few have focused on the area of health assessment needs of parents experiencing perinatal loss. Not many facts are known about the impact of perinatal loss has on the lives of those experiencing it in Malaysia. There is paucity of literature is available with regards to the lives of mothers who have experienced perinatal loss after being discharged from hospital, how do they react to their loss, and what happen to their family relationships with each other following the loss. The present study was aimed to look into psychosocial problems and support obtained following a perinatal loss based on religion and cultural perspectives amongst the Muslims who constitute the largest group in Malaysia. The respondents were questioned on their experience according to four themes: womens’ feeling following perinatal loss, women perception on the role of health care giver during grief, support during grief and decision making.

Psychosocial impact in this study was defined as the entire process precipitated by loss through perinatal death. Perinatal death was defined as death of a foetus during pregnancy at gestational age of 22 weeks and more or birth weight of 500 g and more or during the neonatal period (up to the 28th days). Support was defined in this study as function that prevented or reduced stress in a woman experiencing perinatal loss. The support includes steps to make her feel accepted, respected, reassured that she was cared for and able to communicate freely and shared her experiences and feelings.

## Methods

A qualitative, exploratory and descriptive analysis was used in this study with the objective to explore the psychosocial experience and support following perinatal loss. The researcher explored women need and perceptions following perinatal loss. The respondents were recruited among women attended Universiti Kebangsaan Malaysia Medical Centre (UKMMC) who were recently experienced perinatal loss. In-depth interviews were carried out which lasted from thirty minutes to an hour. Each respondent had several sessions, using triangulation techniques until the objectives were met and the respondent felt comfortable. Respondents were recruited until saturation was reached i.e. until no new information was forthcoming from additional respondents. Data was collected using in-depth unstructured interviews which was sharing their previous memories. As most of the respondents felt uncomfortable with recording, the interviewer noted their comments verbatim and also noted their body language and voice tone (observation). Observations of the respondent’s family members present during the interview session were also noted. Data was analysed through open coding. A focus group discussion (FGD) was arranged among women who had perinatal loss between May 2008 and April 2009. Women selected were chosen from a list of perinatal deaths data reported under rapid reporting of stillbirths and neonatal deaths system. This system compiled deaths of gestational age 22 weeks and above or birth weight 500grams and above. The researchers contacted each selected and volunteered respondent to confirm an appointment at a central place (UKMMC) at an agreed time. Privacy was ensured during the interview. Women were ensured that their participation was entirely voluntarily and they could withdraw from the research at any stage if they wished. The interviews would be stopped if women suffered severe stress during the interview. Permission to conduct the study was received from the UKMMC under specific grant FF-293-2008 and on-going survey was further supported by grant UKMHEJIM- komuniti-04-(2010).

The study population consisted of mother who had experienced perinatal loss and admitted in the maternity unit of the UKMMC. The FGD interviews were conducted between 6 months to 12 months following perinatal loss. Cases with previous medical record of psychiatric illnesses were excluded. A list of women who had experienced perinatal loss and lived within 15 Km radius of the hospital was prepared. Of 15 candidates, only 6 cases volunteered and agreed to participate in this study for the FGD. The researcher also created a conducive environment for mutual trust between the researcher and the respondents. The questions were unstructured and the respondents were asked to describe their experiences in having perinatal loss, dealing with hospital or health staff or with their surroundings. In-depth interview was also carried out among 10 sets of parents within a week following a perinatal loss.

## Results

Table 1 described each respondent’s background based on information gathered from them and hospital record for accuracy of data on causes of deaths, termination offered and gestational age at delivery. Six respondents volunteered to participate for FGD and 10 respondents for in-depth interview. All respondents who participated in this study were Malays of Muslim religious background and lived in urban areas. The age range varied between 23 and 37 years and parity range was between one and five. Data retrieved from hospital record showed that four mothers had perinatal loss within a month after delivery and seven had a macerated stillbirth or fresh stillbirth. Four of sixteen had been classified causes of death due to lethal congenital malformation and two immaturities. Others were classified as unknown. In total, 4 of them had neonatal deaths and another 12 respondents had perinatal loss before birth. Two of the respondents were house wives, four were full time government workers and the others worked in the private sector. All respondents had at least secondary education level qualification. The interval duration from the event of perinatal loss and the interview was 6–12 months for FGD and less than a week for in-depth interview.

**Table 1:** Description of the respondents

**List**	**List of respondents identifier**	**Age (years)**	**Working status**	**Causes of deaths based on Malaysian Modified Wigglesworth classification**	**Gestational age at delivery (weeks)**	**Time bad news informed to mother in relation to delivery**	**Termination of pregnancy offered**
R1	FGD1	28	Government	lethal congenital malformation	28	Prior	yes
R2	FGD2	24	private	unknown fresh stillbirth	37	Prior	no
R3	FGD3	23	private	lethal congenital malformation	32	Prior	yes
R4	FGD4	34	government	normally formed stillbirth	38	During	no
R5	FGD5	37	government	unknown neonatal death	37	Post	no
R6	FGD6	33	private	lethal congenital malformation	29	Prior	yes
R7	ID1	37	Housewife	unknown fresh stillbirth	37	During	No
R8	ID2	38	Housewife	normally formed macerated stillbirth	36	During	No
R9	ID3	28	Private	lethal congenital malformation	32	After	No
R10	ID4	24	Private	immaturity	26	Prior	yes
R11	ID5	34	government	unknown neonatal death	37	Post	no
R12	ID6		private	unknown neonatal death	39	Post	no
R13	ID7	23	private	fresh stillbirth	36	Post	no
R14	ID8	37	government	normally formed macerated stillbirth	34	During	no
R15	ID9	31	private	normally formed macerated stillbirth	32	During	no
R16	ID10	26	government	immaturity	29	Prior	yes

Legend: FGD = focus group discussion. R = respondent. ID = in depth interview.

Results of the in-depth interviews and FGD were described according to respondent’s experience of losses and care received from doctors or midwives during or following delivery. The analysis was grouped into 4 themes: women’s feeling following perinatal loss, women perception on the role of health care giver during grief, support during grief and decision making. Table 2 showed data obtained from the respondents were divided into 4 themes.

**Table 2:** Additional experiences described based on themes explored

**Theme**	**Respondents’ description**
**Theme 1:**	
**Parent feelings on perinatal loss.**	
**Subtheme:**	
Confusion	*Immediately after I delivered Syahmi, ‘I feel my heart stop beating, ‘why my baby Syahmi did not cry? I am looking around at doctor’s face in panic. I noticed that the team assisting my delivery look very busy and in hurry. After few minutes later, the doctor told me that my baby is not doing well and does not look normal . The doctor whispered very softly that the team is trying very hard to survive him. He needs to be transfer to NICU. I felt panic and scared. I wonder what goes wrong’.(R9)*
Feeling of emptiness	*‘During my last antenatal check up in UKMMC, the doctor told me that my baby had died in my womb could be about a week. I had my previous check up at private clinic and the doctor told me that I do not have any problem. Now another doctor told me my baby died. I feel confuse. How it happen within a week? I wonder how the baby will comes out. He already died in my womb. I have carried him almost 7 months and my tummy look big enough and heavy. I don’t like any procedure on me’.(R6)*
Anger	*I asked myself, did I do something wrong? Why did this happen to me?(R6)*
Guilt	*‘Sometimes it’s so annoying hearing other babies crying. But I have nothing. I want to go home. I don’t want to see anybody’.(R15)*
Anxiety over subsequent pregnancy	*I stared at the ultrasound monitor screen and looking at my baby face, body but I don’t see any movement no sign of heart beating as showed by doctor. She was dead. But I am having labour pain and I feel so stress. I am hoping my husband to support me. I am confused and I am scared’.(R14)*
**Theme 2: parent perception of the role of health care giver during grief.**	
Subtheme:	
Lack of communication/information/counselling	*‘I had my last child 5 years ago, she is normal, healthy baby and I delivered her with no problem. I planned for this pregnancy loss. Unfortunately, during my last check up the doctor did a scan and pointing to me and say, ‘Did you see what I see? Look at your baby; it doesn’t look normal with big head and small tiny body, no heart beat seen. Do you feel any baby kicking? In my mind, I knew that for the past few days I hardly feel my baby moving or kicking in my tummy. It never alerts me to sign of danger to my pregnancy outcome. I feel blurred and inside I am blaming myself. What have I done? Why no one remind me that observing baby kick is important?(R1)*
Lack of privacy	*‘After delivered Hidayah, I was pushed to a normal ward. Everybody have baby on their side in the baby cot. I don’t have. I heard their baby cries, it hurt me, and I feel like to shout to them. No one bother me. Is it because I don’t have baby on my side?(R7)*
**Theme 3: support during grief.**	
**Subtheme:**	
Immediate family friend	*I see my son with breathing tube in ventilator. I prayed for a breakthrough. I keep asking my family to pray for my son and I never give up doing this. I knew God will listen and care for me. I feel relieve seeing my friends read Dua and recite Qu’ran for my son. My heart has never stop from asking God to save my son.(R5)*
Religious activity	*‘My family and friends always remind me to be patient and take this as a test from God. They said that whatever problem I have, I must ask Allah to help solving by making a Dua or prayer. Allah is gracious and merciful. I hope Allah will bless me and solve it in the near future’.(R16)*
**Theme 4: decision making**		
	‘*I have to trust my husband. I knew he is strong to face this, supportive, more confident than me and able to plan what the best for us. I don’t want to think anything. I feel so sad. My mind is miserable’.(R3)*	

### Theme 1: Women’s feelings on perinatal loss

#### Confusion

All respondents mentioned that when they were informed about perinatal loss they felt confused and difficult to accept. They did not know what to enquire for further clarification. They said they did not know what to do, and they just listened and nodded their head. What was in their mind was empty and meaningless thinking. Two of these respondents who had experienced loss due to congenital anomaly reported that they felt shocked once they were informed for the first time while scanning during the antenatal check-up at second trimester. When the doctor informed them that the outcome of the pregnancy was not good as their foetus were not well formed or appeared too small for gestational age. They could not really appreciate what this meant by looking at the scan monitor screen or even the printed scan image. Another two mothers who were informed of foetal anomaly did mention that they found it difficult to trust the doctor and hardly understood even though the doctor repeatedly mentioned that their pregnancy would not give a good outcome. Another two mothers with medical problems encountered during pregnancy, and who were offered termination of pregnancy also felt confused at the beginning when the bad news was delivered.

#### Feeling of emptiness

All mothers mentioned that they felt lonely after the perinatal loss. Two of them who experienced intrapartum loss said that even though the doctor had already informed them that the baby may not survive after delivery, they still hoped for a miracle and felt that the baby was still alive. They felt very frustrated and said that their hope gone when saw their babies not moving. One of the mothers said that, even though many months have passed by, they still had a feeling of trying to hide her problem and portrayed a brave picture. Actually, they cried internally and hoped for help from others and wished that somebody could spare time and able to listen theirs’ sadness. They also reported a feeling of distance and loss of life. Sometimes, they woke up thinking their baby was still alive and felt emptiness when they remembered the true situation. Eight out of ten respondents mentioned that they would like to be alone and confine to their own room trying to recollect the event during antenatal and pain during the delivery. They also dreamt as if the loss was untrue. Most respondents said that their tears just come out running like shower unexplained. They described but the memory was freshly felt compared to the experience delivering other children that alive at birth.

#### Anger

Two out of ten mothers from in depth interview felt anger when they received the bad news. They thought that the doctors did not do enough to save their pregnancies even though both of them were informed that problems noted during antenatal screening may affect their pregnancy outcome. They were asked to go to the nearby hospital for follow-up but this was only to confirm that they were going to lose their pregnancy rather than to increase the chances of having a live baby. Another mother detected with a foetal abnormality was detected during pregnancy said that she did not believe the doctor’s explanation. She had tried many traditional practices hoping that the baby would be born normally and survives. Conflicting medical information made her feels unsatisfied and prompted her to seek alternative practices. When the baby was delivered and did not survive, she was angry because no one had been able to convince her enough. Another mother mentioned that even though she been informed accurately on the poor prognosis for the baby after delivery and that it was better to terminate early, she felt angry because of the late decision (after 3 months bad news informed) made by her husband. She had to wait for 3 months until her husband really dared to decide for termination of her pregnancy.

#### Guilt

Women have a feeling of guilt following perinatal loss. They think that it happens because they do not consume appropriate diet and rest enough which make their foetus smaller than the gestational age. They always feel that theirs’ health status unfit to continue the pregnancy. One of the mother who had premature delivery, said that the health nurse did mention that rest was important during pregnancy but she did not bother it and was busy working to finish her job with a full commitment before her expected date of delivery. She did not realize the important of monitoring baby’s kick chart and regular antenatal follow up. She said that if “*I really follow the advice her baby could be stay longer and healthy”.*

#### Anxiety over subsequent pregnancy

The majority of respondents agreed that even after the trauma of having a perinatal loss they still wanted to have another pregnancy. They felt anxious over the outcome of the next pregnancy. One of the mothers who were pregnant at the time of the interview said that:

"*“It is still fresh in my mind how I carried my previous pregnancy and deliver a breathless dead baby. I am afraid it will happen again”.*"

She had regular antenatal follow up and always asked doctors for reassurance that her pregnancy was safe and the baby was normal. She always wanted to see the baby on the ultrasound screen monitor and listen carefully when the doctor explained that the baby was normal, moving and doing well.

### **Theme 2: women perception on the role of health care giver during grief**

#### Lack of communication/information/counselling

Majority of respondents mentioned that not all healthcare staff who dealt with process of delivery really knew and communicated well with them when perinatal loss happened. Most of them tried hard to deliver their baby. However, the respondents felt that the staffs were not concerned with the feeling once the baby was born. The healthcare provider’s attitude was described as trying to avoid from coming regularly and supporting them. At this time they felt that they wanted somebody who could counsel them, advise them on what to do and help them with the unfamiliar processes of discharge, burial and so on.

They felt lonely and did not have anything to read and see or someone to listen and guide them what they should do. They felt that nobody understood their sadness.

"*“No one care about my feeling. Oh God, please help me, I need someone to help me pass through this pain”.*"

They wanted to voice their concern and have their questions answered but felt that the healthcare team was always too busy.

#### Lack of privacy

The respondents recognized the difficulty in providing privacy in a Government hospital but felt that they really wanted it when faced with the trauma of losing a baby. They also reported that they were disturbed with the cry of the baby in the surroundings.

### Theme 3: support during grief

Many of the respondents mentioned that they turned to their children and family to explore ways of living with this unthinkable loss. Their religious practices of reciting the Quran made them feel more comfortable and openly accept faith.

"“*We organized farewell feast for our son and it helped to ease sorrow because when people came over to our house recited Quran and read Dua, we felt calm”*."

#### Immediate family

All respondents agreed that their immediate family members such as their husband and parents helped in consoling them and gave support and advice regarding what was to be done.

#### Friends

Some close friends gave support in term of listening to their feeling. They felt happy if their friends were willing to sit with them even just to listen and understand them. Five of the respondents who participated in this study and who were working mothers, said that their colleagues gave support to them when they went back to work after maternity leave. Respondents said that they normally did not tell everything to their friends. They felt that their friends may not able to understand. They have confident more with older friends. They felt that their loss was different from what people perceived. They still wanted the relationship and feeling of their loss within them. They felt the connection with their perinatal loss. They felt responsibility and eagerness to take care of them. Sometimes respondents felt that they wanted to avoid talking about their loss and sometime they wanted to talk about it.

"“*When my friends visited me I feel that I want to share my experience with them. But it is hard. But sometime I feel unable to do that.”*"

#### Religious activity

All mothers felt that by practicing religious activities they were able to reduce the pain they suffered and make their mind more accepting of the situation. They mentioned that their family members, especially husbands and their own mothers were the strong people who always reminded them to continue practicing religious activities and pray to God. Respondents who had been informed about the small survival chance before the delivery also mentioned that they accepted faith sincerely as a test from God. They agreed that they should give time to their baby the best quality of life and not to regret it.

### Theme 4: decision making

All respondents mentioned that they relied on their husband and family for decision making in the event of termination of the pregnancy. They also depend on their husband or family for making burial plan after perinatal loss. In fact, planning for another pregnancy is also depending on their husband decision. However, sometimes they felt uncomfortable with the decision made. They always asked for more clarification if they felt unsatisfactory.

## Discussion

Perinatal loss has a great impact on any woman that psychosocial support at this time is of great importance. Psychosocial support following perinatal loss does often not address the needs and cultural perspective of Muslim women, despite the fact that Muslims make up 62.1% of the world population [7]. This was the first study conducted in Malaysia on psychosocial impact of mothers experiencing perinatal loss using the qualitative approach focusing on the majority population group in Malaysia. An earlier study using Edinburgh Postnatal Depression Scale (EPDS) as a quantitative approach has mentioned that 53% of Malaysian mothers who suffered perinatal loss were at risk of postnatal depression[13]. It was reported that difference in cultural practices but not specifically described the religious influence on postnatal psychosocial impact [16].

This study has explored Muslims’ mothers’ experience of perinatal loss. Some shared the same experiences and others differed but it could be related to their social background knowledge on outcome of pregnancy. Focus group discussions conducted 6–12 months after perinatal loss helped them to understand their symptoms to live in the present and to think ahead positively. The present study has shown that mothers who experienced perinatal loss felt confusion, feeling emptiness, guilt and anger. Other studies also mentioned that the majority of parents experiencing perinatal loss felt confusion and were confronted with conflicting messages [4,17]. Another study mentioned that people suffering perinatal loss expressed their wish that people would acknowledge their loss, be considerate and sensitive, and lent a listening ear and emotional support [18]. Factors which have been reported to increase the risk of adverse psychological outcomes for parents following a perinatal death include: perceived inadequate social support, traumatic circumstances surrounding the death, difficulties in coping with a crisis in the past, problematic relationships in the nuclear family and the presence of other life crises. Families and friends were described as the main supporters in helping them get back to a normal life. Turton et al. (2001) showed that parents may also be at risk of developing post traumatic stress disorder (PTSD) as a consequence of perinatal loss and this has shed a new perspective on the consequences of perinatal loss [19]. A past study reported 20% of women fulfilled the criteria of PTSD in a pregnancy following a perinatal loss compared to general population incidence of 0.4% to 4.6% [19]. Kavanaugh and Moro (2006) also found that having good emotional support after the stillbirth may be a protective factor [20].

In UKMMC, parents are allowed to see and hold the dead infant and also keep a momento like a photograph. This study has showed that parent feel satisfied with this practice. As poor communication was highlighted by the respondents, it should be studied in detail in future studies, in order to assess how well our health care providers feel when dealing with parents who have suffered perinatal loss. A recent review showed that parents perceived many healthcare provider behaviours to be thoughtless or insensitive [21]. The health care providers should be able to narrow the gap for appropriate communication and recognize personal discomfort when communicating with parents experiencing perinatal loss. This study also showed that, the religious practice improved mothers’ perception of perinatal loss. A strong religious background made them feel better and this was also observed in other earlier studies [22,23].

In practice we found that there were many complaints from bereaved mothers about the lack of support given to them during their grieving process. A study done by Kennel and Klaus (1984) showed that mothers reported that many doctors and midwives seemed to care about delivering the baby but they apparently do not care about the emotional trauma the mother did undergoing [24]. This study also mentioned that the healthcare providers assumed that the baby was small and therefore their loss should not be as great as it would have been if the baby had lived longer [24].

Overall, those experiencing perinatal loss survive through unfamiliar journey even though it seems unbelievable. They have to do such to continue the rest of their lives with others responsibility of taking cares their own health, husband and their own children. The life force within them keeps them going to face future life. Difficulty in thinking and doing of daily activity was commonly affected at an early stage of perinatal loss. Anxiety was common among mothers who experienced losses especially in facing the next pregnancy. Austin and Laedaer (2000) defined anxiety as the psychological consequence of exposure to a real or imagined stress [25]. A study done by Allison et al. (2011) showed a significant association between anxiety and maternal prenatal diagnosis screening for anomaly [26]. The anxiety can be reduced if proper information, counselling and psychological support are offered. In Malaysia, maternal prenatal diagnosis is not a routine practice. However, premarital screening for HIV is compulsory for all Muslims.

In Malaysia, pre-pregnancy clinic for risk assessment screening was offered only for couples who had previous perinatal deaths or anomalies and is done at a specialist hospital. This may create a barrier for parent to access to these services. Another option which may be well accepted nowadays is to use information available in internet to help people calm and heal their losses. Emotional support to cope with the pregnancy loss was also highlighted by previous study [15]. Gold et al. (2011) depicted that message boards helped them surviving through the process of grief by feeling of less loss and they appreciated unique aspects of internet communication such as convenience, access, anonymity, and privacy[27]. A recent study among lesbian and bisexual women through online surveys showed that women participated and expresses their feeling of perinatal loss online [28]. Peel (2010) in her study manage to explore lifestyle change before conception, reaction to loss, emotional recovery from loss and perception on healthcare provider involvement in the pregnancy loss information using online survey. Admittedly, the present study had few limitations such as sample size number as the authors wanted to emphasise the uniformity in conducting interview sessions. Quality of data obtained from interview or discussion will depend on communication between interviewee and the interviewer. The authors did not introduce themselves as healthcare providers. The authors convinced the respondents that they were willing to listen and support of the perinatal loss as a volunteer person. There were no specific services for parent with perinatal loss in this study site that make the authors have difficulty in assigning suitable place to conduct in- depth interview. Unfortunately, when the subjects were asked after 6 months postnatal to participate in focus group discussion, many of them did not turn up either due to inability to accommodate their time for this study or they may not interested in contributing or sharing their experience.

## Conclusion

In conclusion, every mother experiencing of perinatal loss showed some level of psychosocial impact which affected their feelings but the role of health care provider and support obtained was also found to play a major role. Ability to cope with daily tasks after perinatal loss depends on personal grief perception. Being a Muslim woman is not a reason for not having psychosocial distress. In Muslim practice we have to accept faith from God and encourage thinking positively when accounted to any stressful event. Husband, family and friends close to the women are the best person to give this encouragement. Present study was conducted among the Malay ethnicity with Muslim background showed that the decision making was still dependent on the husband and family. If they did not have support either from health care provider, family or the community around them, these psychosocial problems may lead to psychiatric problems such as depression and anxiety over facing with the next pregnancy. This sequel may be prolonged and affecting not only the mother but also her family relationships and her other children.

## Competing interests

Both authors declare that they have no competing interests and non-financial competing interests.

## Author contributions

RS conceived of the study and participated in its design, coordination and drafted the manuscript. HMK helped in focus group discussion as the moderator. Both authors read and approved the final manuscript.

## Pre-publication history

The pre-publication history for this paper can be accessed here:

http://www.biomedcentral.com/1472-6874/12/15/prepub
